# Dissecting Vancomycin-Intermediate Resistance in *Staphylococcus aureus* Using Genome-Wide Association

**DOI:** 10.1093/gbe/evu092

**Published:** 2014-04-30

**Authors:** Md Tauqeer Alam, Robert A. Petit, Emily K. Crispell, Timothy A. Thornton, Karen N. Conneely, Yunxuan Jiang, Sarah W. Satola, Timothy D. Read

**Affiliations:** ^1^Division of Infectious Diseases, Department of Medicine, Emory University School of Medicine; ^2^Atlanta Veterans Affairs Medical Center; ^3^Department of Biostatistics, University of Washington; ^4^Department of Human Genetics, Emory University School of Medicine; ^5^Department of Biostatistics and Bioinformatics, Emory University School of Public Health

**Keywords:** genomics, whole-genome sequencing, phylogeny, bacteria

## Abstract

Vancomycin-intermediate *Staphylococcus aureus* (VISA) is currently defined as having minimal inhibitory concentration (MIC) of 4–8 µg/ml. VISA evolves through changes in multiple genetic loci with at least 16 candidate genes identified in clinical and in vitro-selected VISA strains. We report a whole-genome comparative analysis of 49 vancomycin-sensitive *S. aureus* and 26 VISA strains. Resistance to vancomycin was determined by broth microdilution, Etest, and population analysis profile-area under the curve (PAP-AUC). Genome-wide association studies (GWAS) of 55,977 single-nucleotide polymorphisms identified in one or more strains found one highly significant association (*P* = 8.78E-08) between a nonsynonymous mutation at codon 481 (H481) of the *rpoB* gene and increased vancomycin MIC. Additionally, we used a database of public *S. aureus* genome sequences to identify rare mutations in candidate genes associated with VISA. On the basis of these data, we proposed a preliminary model called ECM+RMCG for the VISA phenotype as a benchmark for future efforts. The model predicted VISA based on the presence of a rare mutation in a set of candidate genes (*walKR*, *vraSR*, *graSR,* and *agrA*) and/or three previously experimentally verified mutations (including the *rpoB* H481 locus) with an accuracy of 81% and a sensitivity of 73%. Further, the level of resistance measured by both Etest and PAP-AUC regressed positively with the number of mutations present in a strain. This study demonstrated 1) the power of GWAS for identifying common genetic variants associated with antibiotic resistance in bacteria and 2) that rare mutations in candidate gene, identified using large genomic data sets, can also be associated with resistance phenotypes.

## Introduction

*Staphylococcus aureus* is one of the most frequent causes of hospital-associated and community-associated infections worldwide. It is responsible for a wide variety of infections, from minor skin lesions to severe life-threatening diseases such as necrotizing pneumonia, osteomyelitis, endocarditis, and septicemia (David and Daum 2010). One of the key challenges in controlling *S. aureus* infections has been the emergence and spread of strains resistant to multiple antibiotics, including penicillin and methicillin (Chambers and DeLeo 2009; Otto 2012). After the recognition of methicillin-resistant *S. aureus* (MRSA) in the late 1950s vancomycin, a glycopeptide antibiotic was adopted as a first-line therapy for strains not responding to β-lactams. A number of rare cases of fully vancomycin-resistant *S. aureus* (minimal inhibitory concentration [MIC] >16 μg/ml) have emerged since 2002 caused by horizontal acquisition of the *vanA* gene on a conjugative transposon (Perichon and Courvalin 2009). However, it is much more common for clinical laboratories around the world to encounter strains with intermediate vancomycin-intermediate *S. aureus* (VISA) and heteroresistance (hVISA) phenotype caused by mutations in the *S. aureus* chromosome (Enright et al. 2002; Chambers and DeLeo 2009; Howden et al. 2010; Otto 2012). VISA is currently defined as a vancomycin MIC of 4–8 μg/ml by reference broth microdilution (BMD) and hVISA as MIC ≤ 2 μg/ml with subpopulations of VISA determined by a plating-based, population analysis profile-area under the curve (PAP-AUC) method (Pfeltz et al. 2001; Satola et al. 2009). The clinical significance of hVISA/VISA strains is debated in the literature, but studies have reported prolonged bacteremia and in some cases treatment failure associated with the phenotype (Ploy et al. 1998; Song et al. 2004; Lulitanond et al. 2009; Khatib et al. 2011).

Although the full VISA phenotype may emerge through multiple mechanisms, it leads to some common phenotypic and physiological changes, such as excess peptidoglycan production, thickened cell wall, reduced autolytic activity, reduced biofilm formation, loss of fitness, and attenuated virulence in the resistant strains (Cui et al. 2000, 2003; Howden et al. 2010). Vancomycin is thought to become trapped in the thickened cell wall and titrated to below lethal concentration (Sieradzki and Tomasz 2003). Whole-genome comparison and candidate gene sequencing approaches have discovered numerous mutations in hVISA/VISA strains compared with baseline vancomycin-sensitive *S. aureus* (VSSA) strains (Mwangi et al. 2007; Gardete et al. 2008; Vidaillac et al. 2013). For simplicity, we will henceforward refer to all non-VSSA phenotypes as VISA, unless specifically referring to a feature of hVISA. Putative VISA candidate genes reported in multiple studies (table 1) were generally associated with cell wall turnover pathways and include global regulators and cell wall synthesis functions. All genes listed in table 1 are core *S. aureus* genes, found in more than 95% *S. aureus* strains. However, only a few specific substitutions within this gene set have been experimentally validated as being responsible for transition from VSSA to VISA by genetic reconstruction and/or complementation (table 1). In this study, we describe an alternative approach to candidate gene sequencing or longitudinal comparison of genomes for the genetic characterization of VISA: A hypothesis-free genome-wide association study (GWAS) and identification of rare mutations was used to model the genetic basis of resistance acquisition in a genotypically diverse group of 75 clinical *S. aureus* strains.

**Table 1:** Candidate Genes Associated with the hVISA/VISA Phenotype in Previous Studies^a^

Gene	N315 Locus Tag	Function	Experimentally Verified Mutations	Reference
*walR*	SA0017	Response regulator	K208R	Howden et al. (2011)
*walK*	SA0018	Sensor histidine kinase	G223D	Howden et al. (2011); Shoji et al. (2011)
			Q371 deletion	
*rpoB*	SA0500	DNA-directed RNA polymerase β subunit	H481Y	Matsuo et al. (2011, 2013); Watanabe et al. (2011)
*sarA*	SA0573	Accessory regulator		
*graR*	SA0614	Response regulator	N197S	Cui et al. (2009); Matsuo et al. (2011)
*graS*	SA0615	Sensor histidine kinase	T136I	Howden et al. (2008)
*vraF*	SA0616	ABC transporter ATP-binding protein		
*vraG*	SA0617	ABC transporter permease		
*clpP*	SA0723	Clp protease proteolytic subunit	144 bp deletion	Shoji et al. (2011)
*stp1* (*PP2C*)	SA1062	Protein phosphatase 2C	Deletions, Q12stop	Passalacqua et al. (2012); Cameron et al. (2012); Renzoni et al. (2011)
*ccpA*	SA1557	Catabolite control protein		Seidl et al. (2006)
*prsA*	SA1659	Peptidylprolyl isomerase		
*vraR*	SA1700	Response regulator		
*vraS*	SA1701	Sensor histidine kinase	I5N, 234stop	Katayama et al. (2009); Cui et al. (2009); Gardete et al. (2012)
*yvqF*	SA1702	Uncharacterized protein	Y220C	Gardete et al. (2012)
*agrA*	SA1844	Accessory gene regulator A		

^a^The list consists of gene identified by comparative whole-genome and candidate gene sequencing studies. For a limited number of studies, there is evidence from genetic reconstruction/complementation studies that specific variants are responsible for elevated vancomycin resistance (fourth column). In these cases, the amino acid change of the variant is listed.

## Materials and Methods

### Bacterial Strains and Phenotypes

Bacterial strains (*N* = 75) used in this study were provided by several sources detailed in supplementary table S1, Supplementary Material online. Clinical strains from multiple body site sources (blood, sputum, and wound) were collected through the Centers for Disease Control and Prevention Active Bacterial Core Surveillance system of the Emerging Infections Program: A nationwide *S. aureus* surveillance program at the University of Iowa Carver College of Medicine, and Emory University Hospital in Atlanta (Klevens et al. 2007; Limbago et al. 2009; Satola et al. 2009, 2011; Richter et al. 2011). The nasal colonization strain was collected as part of a decolonization study by the Washington University School of Medicine in St. Louis (Fritz et al. 2011). Etest MICs were determined using a 0.5 McFarland standard inoculum on Mueller–Hinton Agar plates (Remel, Lenexa, KS) and vancomycin Etest strips (bioMérieux, Durham, NC) according to the manufacturer’s instructions. BMD MICs were determined by the reference method according to Clinical and Laboratory Standards Institute (CLSI) recommendations (CLSI 2012). Because doubling dilutions of 2 and 4 µg/ml were not obtained by this protocol, we grouped these strains based on Etest results. Modified PAP-AUC to detect heteroresistance to vancomycin was performed as described previously (Satola, Farley, et al. 2011). Disk diffusion with 5 µg rifampin Sensi-Disc (BD BBL, Sparks, MD) was performed according to manufacturer’s protocol and CLSI recommendations (CLSI 2012). Pulsed-field gel electrophoresis (PFGE) with *Sma*I restriction endonuclease was performed as described previously (McDougal et al. 2003).

### Whole-Genome Sequencing and Single-Nucleotide Polymorphism Calling

Genomic DNA was isolated from an overnight culture using the Wizard Genomic DNA Purification Kit (Promega, Madison, WI), with the enzyme pretreatment step amended to 50 µg/ml lysostaphin and 500 µg/ml lysozyme in 10 mM Tris-HCl 1 mM EDTA, pH 8.0. Whole-genome shotgun library templates for all 75 strains were made using 1 µg of the genomic DNA, and paired-end sequencing was performed on the Illumina HiSeq 2000 (Illumina Inc., San Diego, CA). Approximately 15 million paired end reads per strain with read lengths of 100 bp, and median coverage of 258× was achieved (supplementary fig. S1, Supplementary Material online). We applied a series of stringent quality-control steps to clean up raw reads before they were used for single-nucleotide polymorphism (SNP) calling. Poor-quality reads were filtered out using PRINSEQ version 0.20.3 in two sequential steps (Schmieder and Edwards 2011). First, we removed any reads with two or more ambiguous bases (Ns) or mean Phred quality score less than 20. Second, low-quality bases (Phred quality score ≤ 19) were trimmed from the 3′-end, and if after the trimming the read length was less than 70 bp or mean Phred quality score of the read was less than 20, they were discarded. The paired end reads passing above quality filters were mapped against *S. aureus* N315 reference genome (GenBank accession number NC_002745) using BWA short read aligner version 0.7.2 (Li and Durbin 2010). The SNPs and insertion–deletions (indels) were called using Genome Analysis ToolKit (GATK) UnifiedGenotyper (McKenna et al. 2010; DePristo et al. 2011). Functional annotations of the SNPs/indels were carried out using an in-house developed *perl* script. SNP/indel was considered true if that position contained at least 10 high-quality reads and 90% or more of them supported alternate allele different from the N315 reference. If an SNP position did not have sufficient coverage or was ambiguous in any one strain, we excluded that SNP from further analysis. The SNPs within the *vraSR*, *graSR*, *walKR*, *vraFG*, *yvqF*, *stp1,* and *rpoB* genes were manually verified by visualizing the alignment files in the Integrated Genome Viewer tool (Thorvaldsdóttir et al. 2013). The output from this workflow was a matrix of 55,977 high-quality SNPs present in the core regions of all 75 sequenced strains. The multilocus sequence type (MLST) genotype of the strains were determined from sequencing read data using the short read sequence typing (SRST) tool (Inouye et al. 2012). The raw sequence reads from the project have been deposited into the National Center for Biotechnology Information (NCBI)’s Sequence Read Archive (SRA) database under the accession number SRP039019. Scripts and data sets used in this study have been made publicly available at https://bitbucket.org/rpetit3/visa-gwas/(last accessed May 8, 2014).

### De Novo Assembly, Annotation, Orthologous Clustering, and Core Genome Phylogeny

The high-quality reads obtained at the above step were assembled into contigs using Velvet de novo genome assembly program version 1.2.06 and then ordered and converted into pseudocontigs using Abacas (Zerbino and Birney 2008; Assefa et al. 2009). The resulting pseudocontigs were annotated using Prokka, a stand-alone tool specifically developed to annotate bacterial genomes (Seemann 2014). The program GET_HOMOLOGUES, which utilizes three popular clustering algorithms- bidirectional best hit (BDBH), COGtriangles and OrthoMCL, was used for identifying and clustering orthologous genes among the strains (Li et al. 2003; Kristensen et al. 2011; Contreras-Moreira and Vinuesa 2013). We evaluated all three algorithms and found no significant difference in the number of orthologous gene clusters predicted by them, so we adopted OrthoMCL algorithm for this study with option -E (expectation value for BLAST alignments) set at 1 e-05 and option -C (minimum percentage of coverage required to call two sequences best hits) set at 75%. Each orthologous protein cluster was individually aligned using Muscle (Edgar 2004) and edited by trimAl (Capella-Gutiérrez et al. 2009) to remove columns with any gap and poorly aligned regions. Finally, all 1,512 core protein alignments were concatenated, leading to the 431,663 amino acids (1,294,989 bp) long superalignment matrix. A neighbor-joining phylogenetic tree was constructed based on the core protein alignment using NEIGHBOR program implemented in PHYLIP (Felsenstein 1989). The genetic distance between sequences was estimated by PROTDIST under JTT amino acid substitution model. Additional editing on the tree was performed using EvolView (Zhang et al. 2012). For orthologous gene clustering and core-genome phylogeny, we also included 49 completed *S. aureus* genome sequences from the NCBI RefSeq database (accession numbers provided in supplementary table S2, Supplementary Material online). The completed genome sequences were reannotated using Prokka as described above, to maintain consistency in our method of predicting genes and other downstream analyses.

### Genome-Wide Association Testing

A number of statistical methods are available for GWAS using whole-genome SNP data (Wu et al. 2011). The basic principle of all these methods is to examine whether there is a statistically significant association between any SNP and the phenotype of interest, without any a priori hypothesis. However, the presence of population structure or cryptic relatedness of samples is a well-known source of spurious association in human studies. Because the phylogenetic relationship between strains depicted in figure 2 is analogous to population structure or cryptic relatedness, we performed our genome-wide association analysis using ROADTRIPS, a recently developed method for genotype–phenotype association tests that uses the genome-wide covariance structure between individuals to account for the presence of unknown population structure or relatedness within the samples (Thornton and McPeek 2010). We also used QROADTRIPS, a new version of ROADTRIPS designed to handle quantitative traits, to test a total of 55,977 high-quality SNPs for association with MIC. Although this approach should rule out any systematic bias in the results due to population structure or any other confounders in the samples, we also calculated the genomic control factor and inspected the resulting quantile–quantile plot for evidence of inflation. A genome-wide association was considered statistically significant if the *P* value for any SNP was less than the Bonferroni-corrected significance threshold (*α* = 0.05/55,977 = 8.9 × 10^−^^7^).

### Genome-Wide Genetic Differentiation

The genetic differentiation (*F*_ST_) value for all 55,977 SNP positions was calculated using the method of Weir and Cockerham (1984) as implemented in an R script available at www.evachan.org (last accessed May 8, 2014). The subpopulations (VSSA and VISA) were ascribed based on Etest result cutoffs.

### Identification of Mutations Enriched by Convergent Evolution to VISA

To identify SNPs enriched by convergent evolution, we used an approach similar to the method described by Farhat et al. (2013). Briefly, we constructed a parsimony tree based on an alignment of core genes and then performed an ancestral sequence reconstruction using the pratchet and ancestral.pars tools from the R phangorn module (Schliep 2011). We then identified mutations that were specifically enriched in branches leading to VISA leaf nodes of the tree compared with VSSA and measured statistical significance using a Fisher’s exact test.

### Identification of Rare Mutations by Comparison to Public *S. aureus* Genome Project Data

We used a database of SNPs called from public *S. aureus* data described in a recent publication (Pishchany et al. 2014). A total of 3,277 raw sequencing project submissions were downloaded from the public NCBI Sequence Read Archive (SRA) on January 10, 2013. These genomes were also mapped against the *S. aureus* strain N315 reference genome and SNPs were called as described above.

### Classification Model Construction

The experimentally confirmed mutations + rare mutations in candidate genes (ECM+RMCG) VISA prediction model was developed by testing combinations of putative genes using an interactive visualization of the project data (https://tread.shinyapps.io/VISA-shiny/, last accessed May 8, 2014). The Random Forest model was constructed using the R package *randomForest* (Liaw and Wiener 2002). A feature table of candidate genes with rare mutations and experimentally confirmed mutations for each strain, along with Etest phenotype was constructed and randomly divided into 47 training strains and 28 test strains. The first iteration of training was run using the parameters mytry = 10 and ntree = 10,000. From this result, six significant features (rpoB H481, SA0017, SA1659, SA0617, SA1702, and SA1700) were selected for a second iteration of the classifier, using mytry = 4 and ntree = 10,000 parameters. The second classifier (*etest_rf_pred2*) was then used to predict the phenotype of the test set. The R script used to create the Random Forests has been made publicly available at https://bitbucket.org/rpetit3/visa-gwas/(last accessed May 8, 2014).

## Results

### Phenotypes and Genotypes of the Sequenced *S. aureus* Strains

We selected a total of 75 clinical strains typed as VSSA and VISA, each category having an approximately balanced spectrum of PFGE genotypes. Fourteen strains were putatively typed as VISA by the CLSI-approved BMD method (CLSI 2012), and 26 were typed VISA by Etest (vancomycin MICs 3–8 µg/ml). According to the PAP-AUC method, 33 strains were identified as hVISA and 14 as VISA (supplementary table S1, Supplementary Material online) (Pfeltz et al. 2001; Satola, Farley, et al. 2011). Genomes were sequenced using Illumina technology with median coverage of 258-fold average nucleotide redundancy (supplementary fig. S1, Supplementary Material online). We obtained multilocus sequence type (MLST) genotypes from the raw sequence data using the SRST algorithm (Inouye et al. 2012). Although there were a number of minor genotypes, the majority of strains were found to be from clonal complex (CC) 5 (51/75, 68%) and CC8 (17/75, ∼23%) (supplementary table S2, Supplementary Material online).

Discrepancies between methods for VISA phenotype definition have hampered standardization between studies (Satola et al. 2009; Satola, Farley, et al. 2011). To compare methods for ascertainment of the VISA phenotype, the vancomycin MIC for all 75 strains (and also several laboratory strains with complete genome sequence already available) was determined using three common methods: BMD, Etest, and PAP-AUC (supplementary table S1, Supplementary Material online). We found that, although there was significant overlap, several strains fell into different categories depending on the test deployed. Using BMD, 61/75 (81.3%) strains had vancomycin MICs ≤ 2µg/ml and were thus defined as VSSA and 14/75 (18.7%) had MIC = 4 µg/ml and were defined as VISA (supplementary table S1, Supplementary Material online). Using Etest, a simple test-strip diffusion assay, 49/75 (65.4%) strains were VSSA and 26/75 (34.6%) were VISA, the latter category overlapping with all 14 BMD-identified VISA. For the PAP-AUC test, considered the gold standard for determination of hVISA (Pfeltz et al. 2001; Satola, Farley, et al. 2011), an area under curve value ≥ 0.90 compared with reference strain Mu3 was defined as heteroresistant. PAP-AUC identified 47/75 (62.6%) strains with a ≥0.90 ratio to Mu3. Of these 47, only 21 (44.7%) were VSSA by both BMD and Etest. Of the 12 strains found to be VISA by Etest but VSSA by BMD, all had borderline MIC of 3 µg/ml, and 10/12 (83.3%) strains were hVISA by PAP-AUC. Even though PAP-AUC is used to determine VSSA or hVISA only and not VISA (Satola, Farley, et al. 2011), for the 14 strains defined as VISA by both BMD and Etest, each strain was given the phenotypic designation of VISA by PAP-AUC as well (supplementary table S1, Supplementary Material online).

### rpoB H481 Is the Predominant Locus Associated with Increased Vancomycin MIC

**Fig. 1.—:**
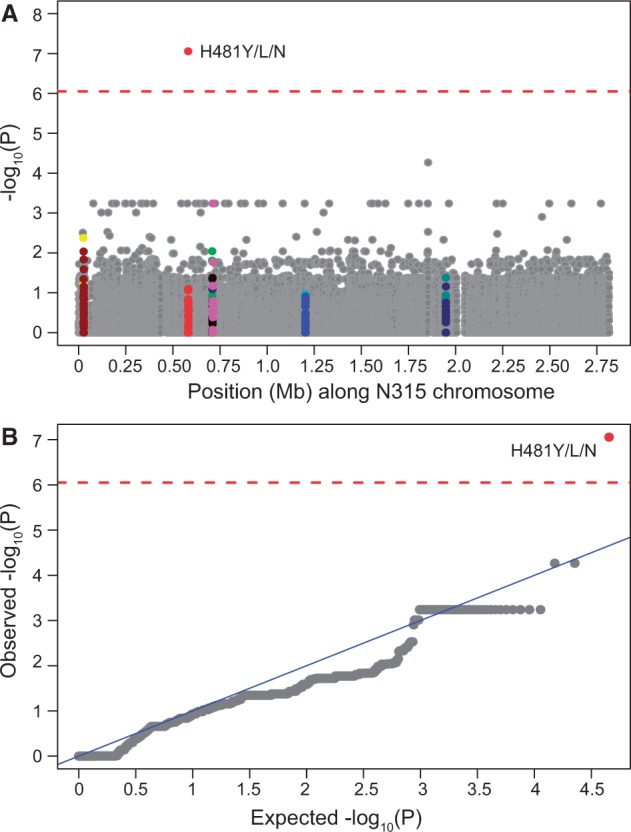
(*A*) Manhattan plot showing the significance of association between 55,977 SNPs and the Etest-based VISA phenotype using QROADTRIPS. The *x* axis shows SNP positions (Mb) in order of N315 reference genome coordinate and the *y* axis shows −log_10_ of the *P* values (−log_10_ [*P*]) resulting from the association test. Each dot in the plot represents an SNP, and SNPs within the most frequently mutated genes *walK* (yellow), *walR* (maroon), *rpoB* (red), *graR* (lime), *graS* (navy), *vraF* (black), *vraG* (pink), *stp1* (aqua), SA1063 (blue), *vraR* (green), *vraS* (blueish-green), and *yvqF* (navy) are highlighted. (*B*) Quantile–quantile (QQ) plot comparing distribution of observed *P* values against expected *P* values distribution under the null hypothesis of no association. If the two distributions are similar, the points should fall roughly on the 45-degree reference line. Genomic control inflation factor = 0.60. The *rpoB* H481 position is indicated on the plot. The horizontal red dashed line in both plots indicates Bonferroni-corrected significance threshold of 0.05 for 55,977 independent tests (−log_10_ [*P*] = 6.05).

**Fig. 2.—:**
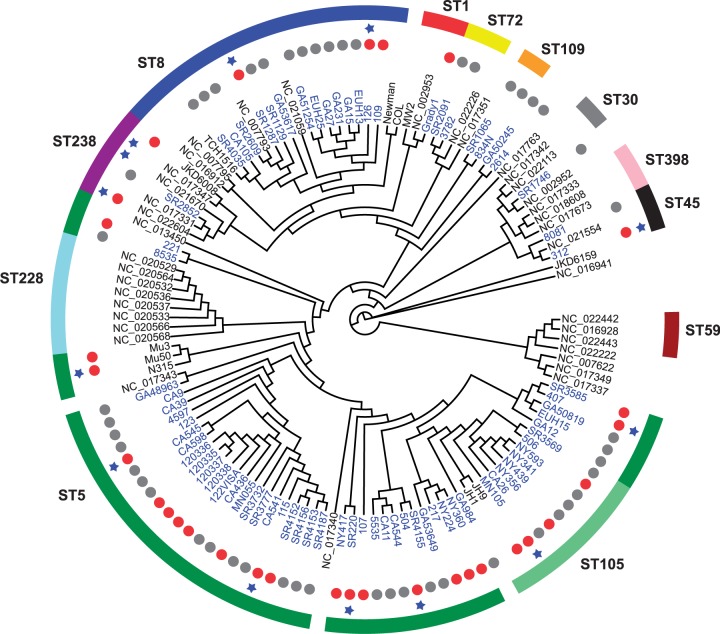
Neighbor-joining cladogram based on 1,512 core protein clusters describing genetic relationships among 124 *Staphylococcus aureus* strains. The 75 strains highlighted as blue text are from this study, whereas the remaining 49 strains are from the NCBI RefSeq database (details provided in supplementary tables S1 and S2, Supplementary Material online). The genomes from the RefSeq database are labeled either by their popular strain name (e.g., COL, Newman, N315, Mu50, and MW2) or accession numbers (those with NC prefix, such as NC_002953). Gray dots represent VSSA strains, red dots represent VISA, and those without dots are strains with unknown vancomycin susceptibility profiles. The blue star indicates strains harboring *rpoB* H481Y/L/N mutation. The MLST type (outer ring) represented by more than two strains is indicated, whereas others are singletons. The MLST types and accession number of all 49 completed genomes are provided in supplementary table S2, Supplementary Material online.

Using the core genome of N315 (ST5; VSSA: MIC < 0.5 µg/ml) as the reference for SNP calling, we obtained a matrix of 55,977 high-quality SNP positions across all 75 strains sequenced in this study. A large number of SNPs were fixed lineage-specific substitutions (i.e., structured by population) that were noninformative in association studies. To identify SNPs within this set that were associated with vancomycin-intermediate resistance we used ROADTRIPS (Thornton and McPeek 2010), a regression-based approach that also accounts for unknown and known population structure in analyses of binary (ROADTRIPS) or continuous (QROADTRIPS) outcomes. We found that no SNP was associated above the Bonferroni-corrected threshold with the binary classification of VSSA/VISA based on Etest results using ROADTRIPs. However, analysis of MIC via QROADTRIPS revealed a strong association between one locus (*rpoB* H481Y/N/L) and an increase in the Etest MIC as a continuous variable (*P* = 8.8 × 10^−^^8^) that surpassed our Bonferroni-corrected threshold of *α* = 8.9 × 10^−^^7^ (fig. 1). The *rpoB* H481 locus was also the only significant association discovered using a permutation test for convergently evolved SNPs found specifically with branches leading to enhanced resistance similar to that recently published by Farhat et al. (2013). Using the same methodology, there were no nonsynonymous SNPs found to be significantly associated with the VSSA phenotype. Seven of 26 strains assigned as VISA by Etest had the H481Y allele, one each carried H481L and H481N mutations, while none of the VSSA strains harbored H481 mutation (supplementary table S3, Supplementary Material online). As shown in the core-protein-based phylogeny, the *rpoB* H481Y/L/N mutation was not restricted to a single genetic background in the VISA strains but was discovered in ST8 (2 strains), ST5 (5 strains), ST105 (1 strains), and ST45 (1 strains) (fig. 2). This was consistent with the hypothesis that intermediate vancomycin resistance evolved independently in each strain as a result of clinical drug exposure.

When we performed association tests using the BMD data resistance phenotypes, the *rpoB* H481 locus was found to be associated with VISA only under categorical classification (supplementary fig. S2*A* and *B*, Supplementary Material online). However, for the PAP-AUC data, we were able to find significant association of *rpoB* H481Y/L/N with only continuous classifier (using QROADTRIPS) and not when strains were binned as VSSA (ratio < 0.9) or hVISA/VISA (≥0.90), using ROADTRIPS (supplementary fig. S2*C* and *D*, Supplementary Material online). Also consistent with our GWA result, only *rpoB* 481 codon position showed elevated Weir and Cockerham *F*_ST_ (*F*_ST_ = 0.200) in our genome-wide *F*_ST_ analysis, whereas *F*_ST_ value for all other loci were extremely low (supplementary fig. S3, Supplementary Material online).

**Fig. 3.—:**
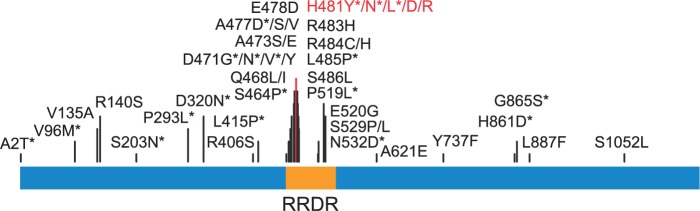
Most frequently described SNPs in the *rpo*B (1,183 amino acids). The SNPs with asterisk were found in our study and in other studies. The RRDR (amino acids 463–550) is highlighted. The H481Y mutation significantly associated with VISA phenotype is shown in red.

The finding of *rpoB* H481 as the sole significant locus in this study also highlighted a relationship between rifampin resistance and exposure to vancomycin in *S. aureus* that had been noted in previous work (Mwangi et al. 2007; Cui et al. 2010; Watanabe et al. 2011; Hafer et al. 2012). SNPs within *rpoB* gene confer rifampin resistance in many bacterial species, including *Escherichia coli*, *Mycobacterium tuberculosis,* and *S. aureus* (Jin and Gross 1988; Musser 1995; Aubry-Damon et al. 1998; O’Neill et al. 2006; Watanabe et al. 2011). In 49 completed *S. aureus* genomes available in the NCBI’s RefSeq database, the *rpoB* H481Y mutation is present in two VISA MRSA strains (Mu50 [ST5] and Z172 [ST239]) where it also confers resistance to rifampin and a third strain Bmb9393 (ST239) whose vancomycin profile is not known but isolated from a bloodstream infection like other intermediate resistance strains (fig. 2) (Hiramatsu et al. 1997; Hiramatsu 1998; Costa et al. 2013; Chen et al. 2013). A total of 17 SNPs were identified in the *rpoB* gene in 75 strains sequenced in this study. Eight of them, however, were concentrated within the rifampin-resistance determining region (RRDR) corresponding to amino acid position 463 to 550 (O’Neill et al. 2006) (fig. 3 and supplementary table S3, Supplementary Material online). Six of the RRDR SNPs (D320N, S464P, D471G/N, A477D, H481Y/N, and P519L) have previously been reported in clinical rifampin-resistant *S. aureus* strains (Aubry-Damon et al. 1998; O’Neill et al. 2006; Mick et al. 2010). When we tested our strains for rifampin resistance using a disk diffusion assay, we found that 11 strains were fully resistant and two were intermediate (supplementary tables S1 and S4, Supplementary Material online). All resistant and intermediate strains had nonsynonymous mutations in the *rpoB* gene. Nine out of 11 fully rifampin-resistant strains and one intermediate strain were VISA by Etest.

### Rare Mutations in Key Candidate Loci Associated with VISA

Other than *rpoB* H481Y, no locus showed a statistically significant association with MIC in our GWA analyses. However, this mutation is not present in all VISA strains, and therefore, the phenotype must be caused by other mutations that are not detectable by association at genome-wide significance levels given the sample size of strains available. We theorized that these mutations were most likely to occur in the group of 16 “candidate” genes for which there were already evidence of involvement with the vancomycin-intermediate resistance from previous genetic studies (table 1 and supplementary table S3, Supplementary Material online). These mutations would be rare in the general *S. aureus* population because the VISA phenotype is uncommon and maintained in the presence of vancomycin selection. We found that the candidate genes contained a range of 0–16 nonsynonymous mutations compared with the N315 reference, similar to the range of diversity of other genes in the genome (fig. 1). We determined whether an SNP was rare by counting the number of times it occurred in a diverse set of 3,277 *S. aureus* genome projects downloaded from the SRA database and mapped against N315. The arbitrary cutoff was set to the minor allele frequency less than or equal to that of VISA SNP *rpoB* H481 (which occurs 81 times in the 3,277 genomes). Using this criterion, we narrowed down the number of candidate SNPs to 77 (table 2). Three of these SNPs (*walK* G223D, *walR* K208R, and *yvqF* Y220C) have been experimentally demonstrated to induce VISA phenotype (Howden et al. 2011; Gardete et al. 2012). None of the strains in our study, however, were found to carry *walK* G223D, *walR* K208R, and *yvqF* Y220C SNPs (table 1 and supplementary table S3, Supplementary Material online). There were no rare SNPs discovered in the *clpP* gene.

**Table 2:** Rare Variants in 16 Candidate Genes Linked to hVISA/VISA^a^

Candidate Genes	Etest		PAP-AUC	
	VSSA (*n* = 49)	VISA (*n* = 26)	VSSA (*n* = 26)	hVISA/VISA (*n* = 49)
*walR*	1	5	0	6
*walK*	6	5	2	9
*rpoB*	8	8	7	9
*sarA*	1	0	1	0
*graR*	0	1	0	1
*graS*	1	1	1	1
*vraF*	2	1	2	1
*vraG*	4	4	3	5
*clpP*	0	0	0	0
*stp1* (*PP2C*)	4	4	4	4
*ccpA*	1	0	0	1
*prsA*	3	0	1	2
*vraR*	1	2	1	2
*vraS*	1	2	1	2
*yvqF*	5	7	4	8
*agrA*	0	1	0	1

^a^The number of rare variants causing nonsynonymous changes (present in <100 out of 3,277 *S. aureus* genomes) for each candidate gene are listed, categorized by whether they are found exclusively in VISA strains (for the Etest-based classification) or hVISA/VISA (PAP-AUC) for the 75 strains sequenced in this study.

We also screened for rare indels within the candidate gene set and found two strains (120338 and 123) with novel deletion mutations in serine/threonine phosphatase 1 (*stp*1) gene. One of them (in strain 120338) was a 3-bp AGT (coding for Serine) nonframeshift deletion at nucleotide position 405. The second (in strain 123) was an 11-bp AAATTACTAGT frameshift deletion at nucleotide position 397 that was predicted to result in early termination of the *stp1* gene at amino acid position 132 (supplementary fig. S4, Supplementary Material online). Recently, indels in the *stp1* gene, different to those found here were linked with reduced vancomycin susceptibility in two clinical VISA strains (Cameron et al. 2012; Passalacqua et al. 2012). One was a 6-bp GAAGAT in-frame insertion that added two new amino acids aspartate (E) and glutamate (D) at amino acid position 17, the other was a single nucleotide deletion and caused premature termination and formation of a truncated 147 amino acids long protein.

Our hypothesis was that these rare SNPs and indels would be important in differentiating VISA strains from VSSA. We investigated the phenotypic definitions by Etest and PAP-AUC, ignoring BMD in this case because the GWAS results suggested that this typing method could not segregate strains with the prominent *rpoB* H481 mutation. Based on the Etest classification, 41 of the 79 rare mutations (77 SNPs and 2 indels) were only found in the 26 VISA strains (table 2). With the exception of *clpP*, *agrA,* and *graR* (the latter two only have one rare SNP each), the a priori hypothesis that rare mutations in a candidate gene result in the VISA phenotype defined by Etest was therefore false. However, 25/26 VISA strains had at least one rare mutation out of the 41 that were in the “VISA-only” class, suggesting that this group of SNPs and indels could be used for effective classification. Interestingly, the single strain missing a candidate mutation (SR4152) was typed as VSSA by BMD and PAP-AUC (supplementary table S3, Supplementary Material online).

The PAP-AUC-based classification placed 52 rare mutations into the “hVISA/VISA only” bracket, including all six SNPs within the *walR* gene. The largest individual gene difference between the typing methodology was for the *walK*: 9/11 SNPs were only associated with vancomycin-intermediate resistance by PAP-AUC, but only 5/11 by Etest (table 2). All 15 strains classed as VISA contained at least one “hVISA/VISA only” mutations but these mutations were found in only 11/31 classed as hVISA (SR4153, 4187, 4035, 3569, 1746, NY439, GA231, CA544, CA11, CA9, and GA53617). The *rpoB* H481 mutation was found in 3/31 strains classed at hVISA and 6/15 classed as VISA (supplementary table S1, Supplementary Material online).

### A Model for VISA Based on Candidate Genes

We developed a simple model for VISA called ECM+RMCG. This classified a strain as VISA if it contained any of the known mutations in table 1 (including the 2 novel *stp*1 indels described here) and/or a rare mutation in the candidate genes *walKR*, *vraSR*, *graSR,* and *agrA*. Rare mutations in other candidate genes were omitted from the model because they either lacked rare loci or had a high-false-positive rate, as defined by Etest (table 2). Using Etest data from the set of 75 genomes sequenced in this study, the sensitivity of the model (true positives/ true positives + false negatives) was 73%, and the accuracy (true positives + true negatives/ all outcomes) was 81%. With PAP-AUC data (classifying a positive result as either hVISA or VISA) the sensitivity 48% and the accuracy was 64%, the differences reflecting the lower threshold for classification as intermediate vancomycin resistance. For comparison to the performance of a formal machine learning method on these data, we developed a Random Forest classifier (Breiman 2001) for VSSA and VISA using rare mutations in candidate genes and experimentally verified mutations as independent features. The model was trained on a randomly selected set of 47 strains and tested on the remaining 28, obtaining an accuracy of 89% and sensitivity for VISA detection of 57%.

**Fig. 4.—:**
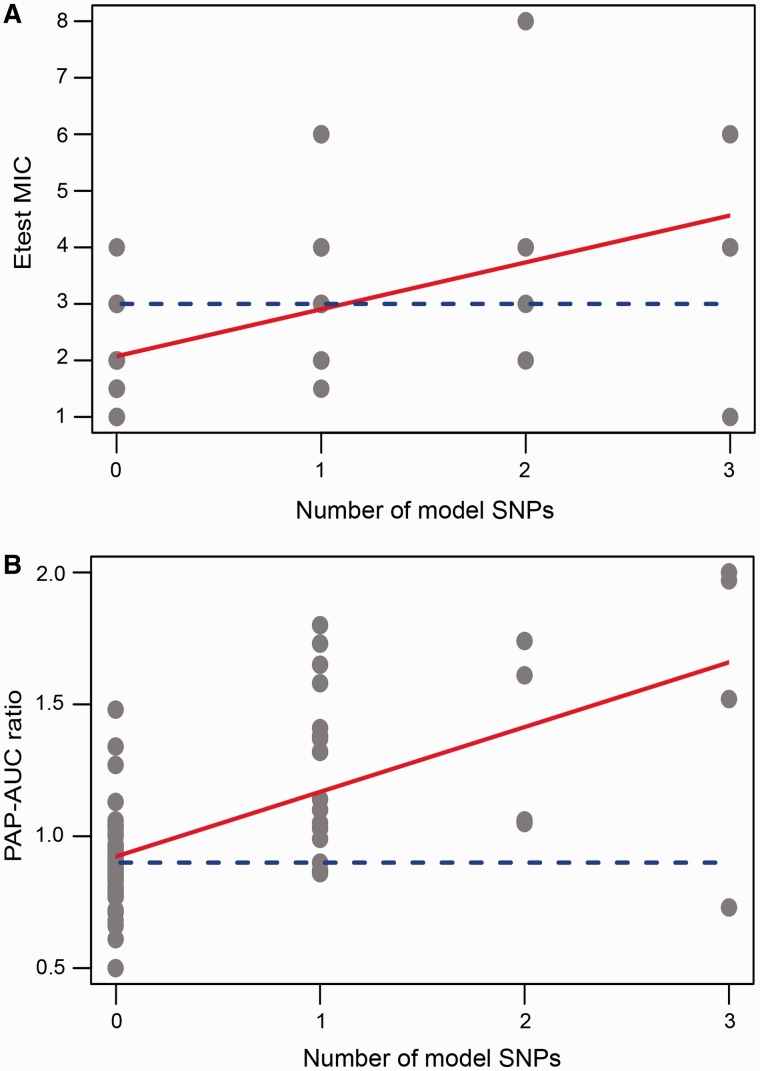
Relationship between the number of ECM+RMCG model loci and vancomycin resistance for each of the 75 strains in the study measured by (*A*) Etest and (*B*) PAP-AUC. The regression lines are shown in red. The dashed blue horizontal line is the threshold for a VISA phenotype, 3 µg/ml in the case of Etest and 0.9 PAP-AUC ratio. In (*A*), the equation of the regression line is *y* = 2.08+0.83*x*. The adjusted *R*^2^ is 0.30 and the *F*-statistic *P* value is 2.52e^−^07. In (*B*), the equation is *y* = 0.92+0.25*x*. The adjusted *R*^2^ is 0.39 and the *F* statistic *P* value is 1.33e^−^09.

Several studies, employing diverse experimental approaches, have suggested a sequential process for VISA evolution, where a population of *S. aureus* under vancomycin selective pressure first evolves into hVISA before transforming into the full-fledged VISA phenotype (Howden et al. 2010). This was demonstrated in the longitudinal comparative genomics study by Mwangi et al. (2007). In confirmation of this hypothesis, we saw a significant regression between the number of ECM+RMCG model loci in a strain and its resistance to vancomycin measured by both PAP-AUC and Etest (*R*^2^ of 0.30 and 0.39, respectively) (fig. 4). When the regression was calculated using all candidate loci, the relationship was more noisy (*R*^2^ of 0.074 and 0.069, respectively for Etest and PAP-AUC) because of the high-false-positive rate associated with rare mutations from some genes (table 2).

## Discussion

The work presented here is the first GWA study, to our knowledge, aimed at investigating drug resistance in *S. aureus*. Previous studies identified VISA-associated mutations pre- and postantibiotic exposure in the course of patient treatment or identified mutations accumulated after laboratory selection for growth on the drug (Passalacqua et al. 2012; Cameron et al. 2012; Vidaillac et al. 2013; Chen et al. 2014). Both these longitudinal approaches are powerful yet suffer disadvantages. The first method requires labor-intensive prospective studies, where many patients who do not subsequently develop intermediate resistance in the course of drug treatment must be screened. Laboratory screening is artificial because it cannot recreate the restrictions on selection imposed by growth inside the host. Recent studies by Vidaillac et al. (2013) and Passalacqua et al. (2012) have demonstrated that the VISA inducing mutations in the laboratory-selected VISA strains are not necessarily the same as in the VISA strains isolated from the patients undergoing vancomycin therapy, even if they are from the same genetic backgrounds. GWA has been used extensively to identify genetic basis of phenotypes in eukaryotes, however, its use in bacteria has been limited. The two main disadvantages often brought forward are the difficulty in obtaining enough cases and controls to obtain statistical power and the constraints on free association between alleles arising from clonal population structure (Falush and Bowden 2006). Despite these caveats, GWAS has been successfully applied to predict SNPs associated with bacterial toxicity and host adaptation (Sheppard et al. 2013; Laabei et al. 2014). Although *S. aureus* known to have a relatively low rate of recombination (Didelot et al. 2012) and the low number of cases and controls used here, we were still able to find a VISA-associated mutation at genome-wide significance levels. This is because vancomycin-intermediate resistance arose through convergent evolution rather than random genetic reassortment. The *rpoB* H481Y/L/N locus was discovered beyond the statistically significant threshold because approximately 35% of VISA strains acquired mutations at this site. We would expect that if we increased the number of cases and controls, other VISA-associated loci occurring at lower frequency may also show statistical significance. The horizontal approach of comparing VISA strains across different cases used in this study would be the easiest way to expand sample size using existing strain collections, because most VISA discovered in clinical laboratories do not have matching recent ancestor VSSA strains. However, the lack of isogenic strains for comparison is a disadvantage when the strain has potentially novel VISA-causing mutations because it is impractical to attempt to screen the large number of candidate loci by current genetic reconstruction techniques.

The importance of the *rpoB* H481 residue in development of VISA was a central finding from this work. Several other studies have also shown that this locus is very frequently mutated in the clinical and laboratory-derived VISA strains (Cui et al. 2010; Watanabe et al. 2011; Hafer et al. 2012; Howden et al. 2014). Given that all the candidate genes are present in all the strains and there is no evidence currently of bias in the rate of mutation at different sites in the *S. aureus* genome, the naïve model would be that each potential VISA mutation would be equally likely to occur for any strain under vancomycin pressure. Epistatic mutations on the genome may have played a role in enriching the *rpoB* locus. Another reason could be that *rpoB* mutants have higher fitness under the clinical conditions encountered by these strains. The exact mechanism for how the *rpoB* H481 mutation confers vancomycin resistance is not understood. Unlike many of the other candidate loci, which are involved specifically in cell wall turnover, *rpoB* is a global regulator, and mutants would be expected to have a large spectrum of pleiotropic phenotypes. An intriguing possibility is that, for a certain subset of patients at least, *rpoB* mutation is preferred over other VISA mutations because of a beneficial secondary phenotype. Further investigation of the basis behind competition between VISA mutations during infection could reveal interesting information about the bacterial/host interaction. It would be of interest to determine experimentally if *rpoB* mutations begin to increase in frequency in *S. aureus* populations within a host as a result of selective pressure before vancomycin treatment or only as a result of the presence of the drug.

The selective advantage for *rpoB* mutation over others could be dual resistance against rifampin and vancomycin (Watanabe et al. 2011; Gao et al. 2013). Most VISA strains with *rpoB* mutations were also reported resistant to rifampin. The first reported VISA strain Mu50 (vancomycin MIC = 8 µg/ml) carried *rpoB* H481Y mutation and was highly resistant to rifampin (MIC > 128 µg/ml), whereas its predecessor Mu3 hVISA did not carry this mutation and was sensitive to rifampin (Matsuo et al. 2011). However, in the United States, over the last decade, rifampin has been infrequently prescribed for *S. aureus* infections. To resolve this issue, future VISA studies would ideally have access to patients’ drug therapy regimen records.

In this study, we showed that the method of ascertainment of vancomycin resistance itself was a major factor in the outcome of analyses. For GWA, *rpoB* H481Y was significantly associated with elevated vancomycin resistance using the values determined by Etest, BMD, and PAP-AUC typing. Further, only the categorical definition of VSSA/VISA-hVISA supplied by PAP-AUC was sufficient for significant association of the locus. Based on the use of rare candidate loci to define vancomycin-intermediate resistance, Etest may be somewhat conservative. On the other hand, PAP-AUC may be prone to false positive assignment, as evidenced by the fact that 11/31 hVISA contained no candidate loci. Future large-scale genomic studies on VISA will likely use Etest, because it is a much simpler and less expensive assay than PAP-AUC, which requires a plating bacteria at multiple dilutions and colony counting. This study shows that there would still be value in performing PAP-AUC on at least a limited number of strains as a consistency check for the phenotype.

The overarching goal of this study was to lay the foundation of a genetic model for VISA that could be used for interrogation of whole-genome sequencing projects. This model would in effect be a definition of the VISA phenotype that would be independent of the experimental method used in a particular laboratory and could be applied to genomics-based epidemiological studies, and ultimately, clinical diagnosis. The problems with defining VISA arise because of variability in the phenotype in the clinical laboratory, variability between phenotyping methods (discussed above), and the large number of rare mutations responsible for altering the sensitivity of the *S. aureus* cell to vancomycin. On any given set of strains, it is possible to create a classifier by focusing narrowly on the mutations encountered (Rishishwar et al. 2014). For example, it would be possible to use the 41 rare variants that are associated only with VISA (table 2) to make a classifier with no false positives and only one false negative. However, the classifier would almost certainly perform far less well on a different set of strains, which would presumably contain common variants such as *rpoB* H481 but also rare variants that had not been seen before. Further genome sequencing studies on much larger sets of VISA strains than performed here are necessary to define all the genes where mutations lead to vancomycin-intermediate resistance. This number of individual mutations putatively associated is likely to be much larger than can be confirmed using genetic reconstruction of point mutations with current laboratory techniques. Therefore, the correct strategy will probably be to confirm experimentally only the mostly frequently discovered mutations and to build a predictive model to estimate the outcome of rarer variants based on knowledge about the genetic context. The approach we used was to combine individual SNPs and indels known to be strongly associated with the phenotype with rare SNPs in candidate genes that we showed to have a low-false-positive rate. The model would generate false positives and negatives but would also be less prone to overfitting. The performance of the ECM+RMCG model described in the results was of approximately similar order to a random forest classifier based on the same data. The purpose of the ECM+RMCG model was to provide a benchmark for comparison of future models based on larger data sets and deeper understanding of the biological basis of VISA. The fact that the sensitivity and accuracy of the model are both under 90% suggests that there is considerable scope for learning useful new knowledge about VISA.

## Supplementary Material

Supplementary tables S1–S4 and figures S1–S4 are available at *Genome Biology and Evolution* online (http://www.gbe.oxfordjournals.org/).

Supplementary Data
