# Supplementary material for: Dissecting Vancomycin-Intermediate Resistance in Staphylococcus aureus Using Genome-Wide Association
Source: Genome Biol Evol. 2014 Apr 30;6(5):1174–85. doi: 10.1093/gbe/evu092 (PMC4040999; doi:10.1093/gbe/evu092)
Supplement: Supplementary Data [file supp_6_5_1174__index.html]

Dissecting Vancomycin-Intermediate Resistance in Staphylococcus aureus Using Genome-Wide Association — Supplementary Data 

# Dissecting Vancomycin-Intermediate Resistance in *Staphylococcus aureus* Using Genome-Wide Association

## Supplementary Data

files

**Files in this Data Supplement:**

- Supplementary Data - doc file
- Supplementary Data - doc file
