# Supplementary material for: Dissecting Vancomycin-Intermediate Resistance in Staphylococcus aureus Using Genome-Wide Association
Source: Genome Biol Evol. 2014 Apr 30;6(5):1174–85. doi: 10.1093/gbe/evu092 (PMC4040999; doi:10.1093/gbe/evu092)
Supplement: Supplementary Data [file supp_evu092_Supplementary_Figures.doc]

Fig S1

Alam et al

**Figure S1**: Box plot showing sequence coverage for 75 *Staphylococcus aureus* strains. The VSSA and VISA isolates are indicated by blue and orange colors, respectively. The dashed blue horizontal line indicates 50x coverage.

Fig S2

Alam et al

A

B

C

D

**Figure S2**: Manhattan plots showing the significance of association between 55,977 SNPs and the BMD-based (panel A and B) and PAP-AUC-based (panel C and D) VISA phenotypes using QROADTRIPS (panel A and C) and ROADTRIPS (panel B and D). The x-axis shows SNP positions (Mb) in increasing order according to the N315 reference genome and the y-axis shows -log_10_ of the P-values [-log_10_ (P)] resulting from the association test. Each dot in the plot represents an SNP and the dashed horizontal line indicates Bonferroni-corrected significance threshold of 0.05 for 55,977 independent tests [-log_10_ (P)=6.05]. The *rpoB* 481 SNP position is indicated.

H481Y/L/N

Fig S3

Alam et al

**
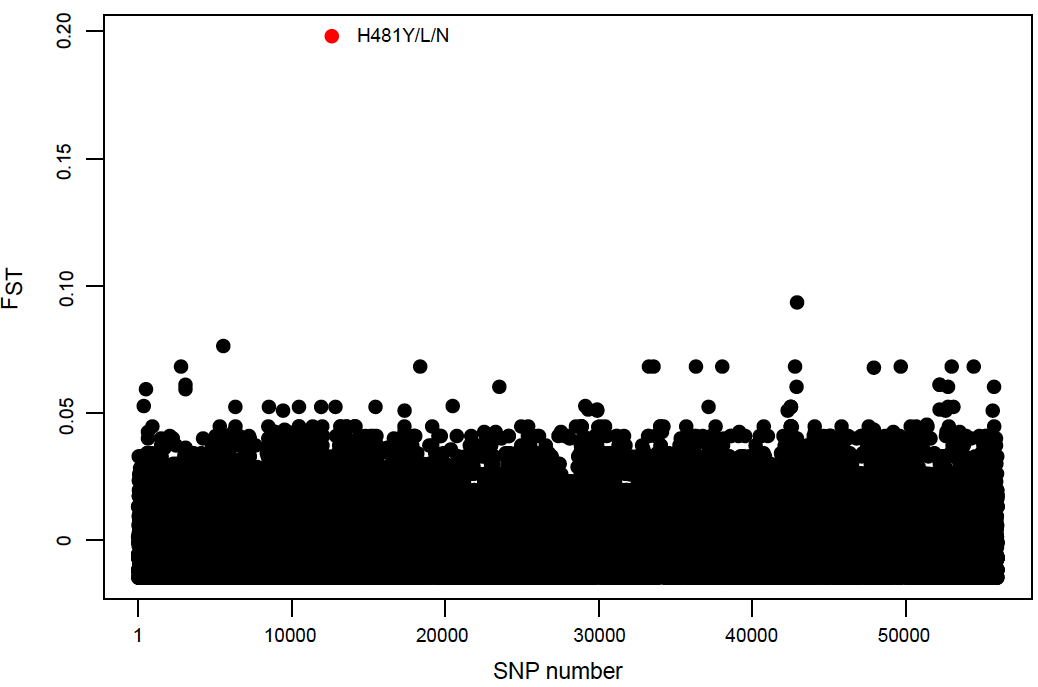
**

**Figure S3.** Genome-wide distribution of Weir and Cockerham F_ST_. Each dot is an SNP, with *rpoB* 481 position highlighted in red. The Etest based classification was adapted to group isolates into VSSA and VISA subpopulations. Similar to GWAS analysis, all 55,977 SNPs were used for F_ST_ analysis.

Fig S4

Alam et al

N315 ATGCTAGAGGCACAATTTTTTACTGATACTGGACAACATAGAGATAAGAATGAAGATGCG 60

120338 ATGCTAGAGGCACAATTTTTTACTGATACTGGACAACATAGAGATAAGAATGAAGATGCG 60

123 ATGCTAGAGGCACAATTTTTTACTGATACTGGACAACATAGAGATAAGAATGAAGATGCG 60

N315 GGTGGTATTTTTTATAATCAAACTAATCAACAACTTTTAGTTCTGTGTGATGGTATGGGC 120

120338 GGTGGTATTTTTTATAATCAAACTAATCAACAACTTTTAGTTCTGTGTGATGGTATGGGC 120

123 GGTGGTATTTTTTATAATCAAACTAATCAACAACTTTTAGTTCTGTGTGATGGTATGGGC 120

N315 GGCCATAAAGCAGGAGAAGTTGCAAGTAAATTTGTTACAGATGAGTTGAAATCCCGTTTT 180

120338 GGCCATAAAGCAGGAGAAGTTGCAAGTAAATTTGTTACAGATGAGTTGAAATCCCGTTTT 180

123 GGCCATAAAGCAGGAGAAGTTGCAAGTAAATTTGTTACAGATGAGTTGAAATCCCGTTTT 180

N315 GAAGCGGAAAATCTTATAGAAGAACATCAAGCTGAAAATTGGTTGCGTAATAATATAAAA 240

120338 GAAGCGGAAAATCTTATAGAAGAACATCAAGCTGAAAATTGGTTGCGTAATAATATAAAA 240

123 GAAGCGGAAAATCTTATAGAAGAACATCAAGCTGAAAATTGGTTGCGTAATAATATAAAA 240

N315 GATATAAATTTTCAGTTATATCACTATGCACAAGAAAATGCAGAATATAAAGGTATGGGT 300

120338 GATATAAATTTTCAGTTATATCACTATGCACAAGAAAATGCAGAATATAAAGGTATGGGT 300

123 GATATAAATTTTCAGTTATATCACTATGCACAAGAAAATGCAGAATATAAAGGTATGGGT 300

N315 ACAACATGTGTTTGTGCACTTGTTTTTGAAAAATCAGTTGTGATAGCAAATGTCGGTGAT 360

120338 ACAACATGTGTTTGTGCACTTGTTTTTGAAAAATCAGTTGTGATAGCAAATGTCGGTGAT 360

123 ACAACATGTGTTTGTGCACTTGTTTTTGAAAAATCAGTTGTGATAGCAAATGTCGGTGAT 360

N315 TCTAGAGCCTATGTTATTAATAGTAGACAAATTGAACAAATTACTAGTGATCACTCATTT 420

120338 TCTAGAGCCTATGTTATTAATAGTAGACAAATTGAACAAATTACT---GATCACTCATTT 409

123 TCTAGAGCCTATGTTATTAATAGTAGACAAATTGAAC-----------GATCACTCATTT 417

N315 GTTAATCATCTTGTTTTAACGGGTCAAATTACGCCGGAAGAAGCATTTACACATCCACAA 480

120338 GTTAATCATCTTGTTTTAACGGGTCAAATTACGCCGGAAGAAGCATTTACACATCCACAA 469

123 GTTAATCATCTTGTTTTAACGGGTCAAATTACGCCGGAAGAAGCATTTACACATCCACAA 477

N315 CGTAATATTATTACGAAGGTGATGGGCACAGATAAACGTGTGAGTCCAGATTTGTTTATT 540

120338 CGTAATATTATTACGAAGGTGATGGGCACAGATAAACGTGTGAGTCCAGATTTGTTTATT 529

123 CGTAATATTATTACGAAGGTGATGGGCACAGATAAACGTGTGAGTCCAGATTTGTTTATT 537

N315 AAGCGATTAAATTTTTATGATTATTTATTATTAAATTCAGATGGATTAACTGATTATGTT 600

120338 AAGCGATTAAATTTTTATGATTATTTATTATTAAATTCAGATGGATTAACTGATTATGTT 589

123 AAGCGATTAAATTTTTATGATTATTTATTATTAAATTCAGATGGATTAACTGATTATGTT 597

N315 AAAGACAATGAAATTAAGCGTTTGTTAGTAAAAGAAGGTACAATAGAAGATCATGGTGAT 660

120338 AAAGACAATGAAATTAAGCGTTTGTTAGTAAAAGAAGGTACAATAGAAGATCATGGTGAT 649

123 AAAGACAATGAAATTAAGCGTTTGTTAGTAAAAGAAGGTACAATAGAAGATCATGGTGAT 657

N315 CAATTAATGCAATTGGCATTAGATAACCATTCGAAAGATAACGTTACTTTCATACTCGCG 720

120338 CAATTAATGCAATTGGCATTAGATAACCATTCGAAAGATAACGTTACTTTCATACTCGCG 709

123 CAATTAATGCAATTGGCATTAGATAACCATTCGAAAGATAACGTTACTTTCATACTCGCG 717

N315 GCTATTGAAGGTGATAAAGTATGA 744

120338 GCTATTGAAGGTGATAAAGTATGA 733

123 GCTATTGAAGGTGATAAAGTATGA 741

**Figure S4.** Sequence alignment of the *stp*1 gene showing non-frameshift (isolate 120338) and frameshift (isolate 123) deletions in two VISA isolates. The alternate codons are underlined to show the right reading frame. The stop codon resulted due to frameshift mutation in isolate 123 is shown in red.
