# Supplementary material for: Dissecting Vancomycin-Intermediate Resistance in Staphylococcus aureus Using Genome-Wide Association
Source: Genome Biol Evol. 2014 Apr 30;6(5):1174–85. doi: 10.1093/gbe/evu092 (PMC4040999; doi:10.1093/gbe/evu092)
Supplement: Supplementary Data [file supp_evu092_Supplementary_Tables.doc]

**Table S1:** Details of *Staphylococcus aureus* strains analyzed in this study

| **S. No.** | **Strain ID** | **Vancomycin MIC (µg/ml)** | | **PAP-AUC** | **Phenotype^e^** | **Rifampin**  **Disc Diffusion** | | **PFGE Type** | **ST** | **CC** | **Body Site Source** | **State** | **Reference/Source** |
| --- | --- | --- | --- | --- | --- | --- | --- | --- | --- | --- | --- | --- | --- |
|  |  | **BMD** | **Etest** |  |  | **Zone (mm)** | **Phenotype^f^** |  |  |  |  |  |  |
| 1 | 2614 | ≤2^d^ | 1 | 0.73 | 0-0-0 | ND | ND | Group D | 12 | 5 | N/A | N/A | CDC |
| 2 | 8081 | ≤2^d^ | 1 | 0.71 | 0-0-0 | ND | ND | USA600 | 45 | 45 | N/A | N/A | CDC |
| 3 | EUH13 | 1 | 1 | 0.5 | 0-0-0 | 35 | S | NA | 8 | 8 | Blood | GA | (Satola et al. 2009) |
| 4 | EUH15 | 1 | 1 | 0.61 | 0-0-0 | 35 | S | USA100 | 5 | 5 | Blood | GA | (Satola et al. 2009) |
| 5 | EUH25 | 1 | 1 | 0.66 | 0-0-0 | 33 | S | USA500/Iberian | 8 | 8 | Blood | GA | (Satola et al. 2009) |
| 6 | 3782 | ≤2^d^ | 1.5 | 0.79 | 0-0-0 | ND | ND | USA700 | 72 | 8 | N/A | N/A | CDC |
| 7 | 4597 | ≤2^d^ | 1.5 | 0.84 | 0-0-0 | ND | ND | USA100 | 5 | 5 | Blood | N/A | CDC |
| 8 | 5535 | ≤2^d^ | 1.5 | 0.85 | 0-0-0 | ND | ND | USA100 | 5 | 5 | Blood | N/A | CDC |
| 9 | 834N | ≤2^d^ | 1.5 | 0.77 | 0-0-0 | 31 | S | Group A | 109 | 9 | Nasal Colonization | MO | (Fritz et al. 2011) |
| 10 | 8535 | ≤2^d^ | 1.5 | 0.8 | 0-0-0 | ND | ND | USA800 | 5 | 5 | N/A | N/A | CDC |
| 11 | GA48963 | ≤2^d^ | 1.5 | 0.81 | 0-0-0 | 35 | S | USA100 | 5 | 5 | Blood | GA | GA EIP |
| 12 | GA50819 | ≤2^d^ | 1.5 | 0.79 | 0-0-0 | 32 | S | USA100 | 5 | 5 | Blood | GA | GA EIP |
| 13 | GA53617 | ≤2^d^ | 1.5 | 0.77 | 0-0-0 | 30 | S | USA300 | 8 | 8 | Blood | GA | GA EIP |
| 14 | CA598 | 2 | 1.5 | 1.06 | 0-0-1 | 34 | S | USA100 | 5 | 5 | Joint | CA | (Satola et al. 2011) |
| 15 | NY593 | 2 | 1.5 | 0.9 | 0-0-1 | 35 | S | USA100 | 105 | 5 | Blood | NY | (Satola et al. 2011) |
| 16 | CA26 | 2 | 2 | 0.68 | 0-0-0 | 32 | S | USA100 | 105 | 5 | Blood | CA | (Satola et al. 2011) |
| 17 | CA541 | 2 | 2 | 0.87 | 0-0-0 | 30 | S | USA100 | 5 | 5 | Blood | CA | (Satola et al. 2011) |
| 18 | GA27 | 2 | 2 | 0.86 | 0-0-0 | 30 | S | USA500/Iberian | 8 | 8 | Blood | GA | (Satola et al. 2011) |
| 19 | GA50245 | ≤2^d^ | 2 | 0.83 | 0-0-0 | 30 | S | USA400 | 97 | 97 | Blood | GA | GA EIP |
| 20 | GA51254 | ≤2^d^ | 2 | 0.77 | 0-0-0 | 35 | S | USA500 | 8 | 8 | Blood | GA | GA EIP |
| 21 | GA53649 | ≤2^d^ | 2 | 0.85 | 0-0-0 | 32 | S | USA100 | 5 | 5 | Blood | GA | GA EIP |
| 22 | SR1065 | ≤2^d^ | 2 | 0.88 | 0-0-0 | 30 | S | Group A | 109 | 9 | Tracheal aspirate | OH | (Richter et al. 2011) |
| 23 | SR1129 | ≤2^d^ | 2 | 0.86 | 0-0-0 | 32 | S | USA300 | 8 | 8 | Wound/Abscess | TX | (Richter et al. 2011) |
| 24 | SR2609 | ≤2^d^ | 2 | 0.89 | 0-0-0 | 0 | R | USA300 | 8 | 8 | Blood | WA | (Richter et al. 2011) |
| 25 | SR2852 | ≤2^d^ | 2 | 0.86 | 0-0-0 | 37 | S | Brazilian | 239 | 8 | Blood | CA | (Richter et al. 2011) |
| 26 | SR4155 | ≤2^d^ | 2 | 0.89 | 0-0-0 | 30 | S | USA100 | 5 | 5 | Sputum | AZ | (Richter et al. 2011) |
| 27 | SR4156 | ≤2^d^ | 2 | 0.88 | 0-0-0 | 30 | S | USA100 | 5 | 5 | Blood | AZ | (Richter et al. 2011) |
| 28 | 120336 | 1 | 2 | 0.72 | 0-0-0 | 31 | S | NA | 5 | 5 | Blood | N/A | CDC |
| 29 | CA105 | 2 | 2 | 0.92 | 0-0-1 | 30 | S | USA300 | 8 | 8 | Blood | CA | (Satola et al. 2011) |
| 30 | CA11 | 2 | 2 | 1.01 | 0-0-1 | 35 | S | USA100 | 5 | 5 | Blood | CA | (Satola et al. 2011) |
| 31 | CA39 | 2 | 2 | 0.95 | 0-0-1 | 33 | S | USA100 | 5 | 5 | Blood | CA | (Satola et al. 2011) |
| 32 | CA436 | 2 | 2 | 0.99 | 0-0-1 | 32 | S | USA100 | 5 | 5 | Blood | CA | (Satola et al. 2011) |
| 33 | CA544 | 2 | 2 | 1.34 | 0-0-1 | 32 | S | USA100 | 5 | 5 | Blood | CA | (Satola et al. 2011) |
| 34 | CA545 | 2 | 2 | 1.04 | 0-0-1 | 34 | S | USA100 | 5 | 5 | Joint | CA | (Satola et al. 2011) |
| 35 | CA9 | 2 | 2 | 1.03 | 0-0-1 | 35 | S | USA100 | 5 | 5 | Blood | CA | (Satola et al. 2011) |
| 36 | GA12 | 2 | 2 | 0.92 | 0-0-1 | 32 | S | USA100 | 5 | 5 | Blood | GA | (Satola et al. 2011) |
| 37 | GA15 | 2 | 2 | 0.92 | 0-0-1 | 33 | S | USA500/Iberian | 8 | 8 | Blood | GA | (Satola et al. 2011) |
| 38 | GA231 | 2 | 2 | 0.93 | 0-0-1 | 27 | S | USA500/Iberian | 8 | 8 | Blood | GA | (Satola et al. 2011) |
| 39 | GA984 | 2 | 2 | 0.9 | 0-0-1 | 16 | R | USA100 | 632 | 5 | Blood | GA | (Satola et al. 2011) |
| 40 | MN055 | 2 | 2 | 0.91 | 0-0-1 | 36 | S | USA100 | 5 | 5 | Blood | MN | (Satola et al. 2011) |
| 41 | NY341 | 2 | 2 | 0.91 | 0-0-1 | 34 | S | USA100 | 105 | 5 | Blood | NY | (Satola et al. 2011) |
| 42 | NY439 | 2 | 2 | 0.95 | 0-0-1 | 30 | S | USA100 | 105 | 5 | Blood | NY | (Satola et al. 2011) |
| 43 | SR1746 | ≤2^d^ | 2 | 1 | 0-0-1 | 31 | S | USA200 | 34slv | - | Other Sterile Site | NY | (Richter et al. 2011) |
| 44 | SR2091 | ≤2^d^ | 2 | 1.05 | 0-0-1 | 30 | S | USA700 | 72 | 8 | Wound/Abscess | NY | (Richter et al. 2011) |
| 45 | SR3569 | ≤2^d^ | 2 | 0.91 | 0-0-1 | 37 | S | USA400 | 105 | 5 | Wound/Abscess | NY | (Richter et al. 2011) |
| 46 | SR3777 | ≤2^d^ | 2 | 1.1 | 0-0-1 | 37 | S | USA100 | 5 | 5 | Blood | MO | (Richter et al. 2011) |
| 47 | SR4035 | ≤2^d^ | 2 | 1.04 | 0-0-1 | 17 | I | USA300 | 8 | 8 | Blood | AL | (Richter et al. 2011) |
| 48 | SR4153 | ≤2^d^ | 2 | 0.96 | 0-0-1 | 35 | S | USA100 | 5 | 5 | Blood | AZ | (Richter et al. 2011) |
| 49 | SR4187 | ≤2^d^ | 2 | 0.93 | 0-0-1 | 30 | S | USA100 | 5 | 5 | Blood | AZ | (Richter et al. 2011) |
| 50 | SR3585 | ≤2^d^ | 3 | 0.87 | 0-2-0 | 35 | S | USA100 | 5 | 5 | Tissue | NY | (Richter et al. 2011) |
| 51 | SR4152 | ≤2^d^ | 3 | 0.87 | 0-2-0 | 30 | S | USA100 | 5 | 5 | Blood | AZ | (Richter et al. 2011) |
| 52 | MN105^a^ | 2 | 3 | 1.05 | 0-2-1 | 0 | R | USA100 | 105 | 5 | Blood | MN | (Satola et al. 2011) |
| 53 | NY224 | 2 | 3 | 1.27 | 0-2-1 | 37 | S | USA100 | 5 | 5 | Blood | NY | (Satola et al. 2011) |
| 54 | NY356 | 2 | 3 | 1.37 | 0-2-1 | 35 | S | USA100 | 105 | 5 | Blood | NY | (Satola et al. 2011) |
| 55 | NY360 | 2 | 3 | 1.38 | 0-2-1 | 34 | S | USA100 | 5 | 5 | Bone | NY | (Satola et al. 2011) |
| 56 | NY417 | 2 | 3 | 1.48 | 0-2-1 | 39 | S | USA100 | 5 | 5 | Blood | NY | (Satola et al. 2011) |
| 57 | SR1287^a^ | ≤2^d^ | 3 | 1.14 | 0-2-1 | 0 | R | USA300 | 8 | 8 | Blood | CA | (Richter et al. 2011) |
| 58 | SR220^b^ | ≤2^d^ | 3 | 1.03 | 0-2-1 | 13 | R | USA100 | 5 | 5 | Sputum | GA | (Richter et al. 2011) |
| 59 | SR3732 | ≤2^d^ | 3 | 1.06 | 0-2-1 | 32 | S | USA100 | 5 | 5 | Blood | MO | (Richter et al. 2011) |
| 60 | 120335 | 2 | 3 | 0.96 | 0-2-1 | 35 | S | NA | 5 | 5 | N/A | N/A | CDC |
| 61 | 120337 | 2 | 3 | 0.97 | 0-2-1 | 30 | S | NA | 5 | 5 | N/A | N/A | CDC |
| 62 | 407^a^ | 4 | 3 | 1.73 | 2-2-2 | 0 | R | USA100 | 5 | 5 | N/A | N/A | CDC |
| 63 | Grady1 | 4 | 3 | 1.32 | 2-2-2 | 32 | S | Group D | 1 | 1 | Blood | GA | CDC |
| 64 | 107 | 4 | 4 | 1.52 | 2-2-2 | 37 | S | USA100 | 5 | 5 | N/A | N/A | CDC |
| 65 | 115^c^ | 4 | 4 | 1.74 | 2-2-2 | 17 | I | USA100 | 5 | 5 | N/A | N/A | CDC |
| 66 | 122VISA | 4 | 4 | 1.13 | 2-2-2 | 38 | S | NA | 5 | 5 | N/A | N/A | CDC |
| 67 | 126^a^ | 4 | 4 | 1.65 | 2-2-2 | 0 | R | USA500/Iberian | 8 | 8 | N/A | N/A | CDC |
| 68 | 217 | 4 | 4 | 1.61 | 2-2-2 | 30 | S | USA100 | 5 | 5 | N/A | N/A | CDC |
| 69 | 221 | 4 | 4 | 1.41 | 2-2-2 | 21 | S | USA800 | 5 | 5 | N/A | N/A | CDC |
| 70 | 312^a^ | 4 | 4 | 1.97 | 2-2-2 | 0 | R | USA600 | 45 | 45 | N/A | N/A | CDC |
| 71 | 506 | 4 | 4 | 1.32 | 2-2-2 | 35 | S | USA100 | 105 | 5 | N/A | N/A | CDC |
| 72 | 109 | 4 | 6 | 1.8 | 2-2-2 | 10 | R | USA500 | 8 | 8 | N/A | N/A | CDC |
| 73 | 123^a^ | 4 | 6 | 2 | 2-2-2 | 0 | R | USA100 | 5 | 5 | N/A | N/A | CDC |
| 74 | 504^a^ | 4 | 6 | 1.58 | 2-2-2 | 0 | R | USA100 | 5 | 5 | N/A | N/A | CDC |
| 75 | 120338 | 4 | 8 | NA | 2-2-2 | 40 | S | NA | 5 | 5 | N/A | N/A | CDC |

^a^Strain with rpoB H481Y mutation.

^b^Strain with rpoB H481L mutation.

^c^Strain with rpoB H481N mutation.

^d^MIC from BMD not known; reported simply as susceptible (VSSA) or ≤ 2 µg/ml.

^e^Phenotype indicates VSSA (0), hVISA (1) and VISA (2) by BMD, Etest and PAP-AUC ratio to Mu3, respectively. For example, the phenotype 0-0-0 means the strain is a VSSA by all three methods, 0-0-1 means the strain is VSSA by BMD and Etest, and hVISA by PAP-AUC method, and 2-2-2 means the strains is VISA by all three methods. PAP-AUC (≥ 0.90 ratio to hVISA strain Mu3) was used to determine VSSA or hVISA only and not VISA. However, if a strain was confirmed VISA by both BMD and Etest, the strain was given the phenotypic designation 2-2-2 or VISA by all three methods.

^f^R (resistant): ≤ 16 mm, I (intermediate): 17-19 mm, S (susceptible): ≥ 20 mm, ND = Not determined

EIP, Emerging Infections Program

**References**

Baba T et al. 2002. Genome and virulence determinants of high virulence community-acquired MRSA. Lancet. 359:1819–1827.

Baba T, Bae T, Schneewind O, Takeuchi F, Hiramatsu K. 2008. Genome sequence of *Staphylococcus aureus* strain Newman and comparative analysis of staphylococcal genomes: polymorphism and evolution of two major pathogenicity islands. J. Bacteriol. 190:300–310. doi: 10.1128/JB.01000-07.

Chen C-J et al. 2013a. Characterization and comparison of 2 distinct epidemic community-associated methicillin-resistant *Staphylococcus aureus* clones of ST59 lineage. PLoS ONE. 8:e63210. doi: 10.1371/journal.pone.0063210.

Chen F, Lauderdale T, Wang L, Huang I. 2013b. Complete genome sequence of *Staphylococcus aureus* Z172, a vancomycin-intermediate and daptomycin-nonsusceptible methicillin-resistant strain isolated in Taiwan. Genome Announc. 1:e01011–13. doi: 10.1128/genomeA.01011-13.

Chen Y, Chatterjee SS, Porcella SF, Yu Y-S, Otto M. 2013c. Complete genome sequence of a Pantón-Valentine leukocidin-negative community-associated methicillin-resistant *Staphylococcus aureus* strain of sequence type 72 from Korea. PLoS ONE. 8:e72803. doi: 10.1371/journal.pone.0072803.

Chua K et al. 2010. Complete genome sequence of *Staphylococcus aureus* strain JKD6159, a unique Australian clone of ST93-IV community methicillin-resistant *Staphylococcus aureus*. J. Bacteriol. 192:5556–5557. doi: 10.1128/JB.00878-10.

Costa MOC et al. 2013. Complete genome sequence of a variant of the methicillin-resistant *Staphylococcus aureus* ST239 lineage, strain BMB9393, displaying superior ability to accumulate ica-independent biofilm. Genome Announc. 1:e00576–13. doi: 10.1128/genomeA.00576-13.

Diep BA et al. 2006. Complete genome sequence of USA300, an epidemic clone of community-acquired meticillin-resistant *Staphylococcus aureus*. The Lancet. 367:731–739.

Fritz SA et al. 2011. Effectiveness of measures to eradicate *Staphylococcus aureus* carriage in patients with community-associated skin and soft-tissue infections: a randomized trial. Infect Control Hosp Epidemiol. 32:872–880. doi: 10.1086/661285.

García-Álvarez L et al. 2011. Meticillin-resistant *Staphylococcus aureus* with a novel mecA homologue in human and bovine populations in the UK and Denmark: a descriptive study. Lancet Infect Dis. 11:595–603. doi: 10.1016/S1473-3099(11)70126-8.

Gill SR et al. 2005. Insights on evolution of virulence and resistance from the complete genome analysis of an early methicillin-resistant *Staphylococcus aureus* strain and a biofilm-producing methicillin-resistant *Staphylococcus epidermidis* strain. J. Bacteriol. 187:2426–2438. doi: 10.1128/JB.187.7.2426-2438.2005.

Gillaspy AF et al. 2006. The *Staphylococcus aureus* NCTC 8325 genome. In Gram-Positive Pathogens, pp. 381–412. Edited by V. A. Fischetti, R. P. Novick, J. J. Ferretti, D. A. Portnoy & J. I. Rood. Washington, DC: American Society for Microbiology.

Golding GR et al. 2012. Whole-genome sequence of livestock-associated st398 methicillin-resistant *Staphylococcus aureus* isolated from humans in Canada. J. Bacteriol. 194:6627–6628. doi: 10.1128/JB.01680-12.

Guinane CM et al. 2010. Evolutionary genomics of *Staphylococcus aureus* reveals insights into the origin and molecular basis of ruminant host adaptation. Genome Biology and Evolution. 2:454–466. doi: 10.1093/gbe/evq031.

Herron-Olson L, Fitzgerald JR, Musser JM, Kapur V. 2007. Molecular correlates of host specialization in *Staphylococcus aureus*. PLoS ONE. 2:e1120. doi: 10.1371/journal.pone.0001120.

Highlander SK et al. 2007. Subtle genetic changes enhance virulence of methicillin resistant and sensitive *Staphylococcus aureus*. BMC Microbiol. 7:99. doi: 10.1186/1471-2180-7-99.

Holden MTG et al. 2004. Complete genomes of two clinical *Staphylococcus aureus* strains: evidence for the rapid evolution of virulence and drug resistance. Proc. Natl. Acad. Sci. U.S.A. 101:9786–9791. doi: 10.1073/pnas.0402521101.

Holden MTG et al. 2010. Genome sequence of a recently emerged, highly transmissible, multi-antibiotic- and antiseptic-resistant variant of methicillin-resistant *Staphylococcus aureus* , sequence type 239 (TW). J. Bacteriol. 192:888–892.

Holt DC et al. 2011. A very early-branching *Staphylococcus aureus* lineage lacking the carotenoid pigment staphyloxanthin. Genome Biology and Evolution. 3:881–895. doi: 10.1093/gbe/evr078.

Howden BP et al. 2010. Complete genome sequence of Staphylococcus aureus strain JKD6008, an ST239 clone of methicillin-resistant Staphylococcus aureus with intermediate-level vancomycin resistance. J. Bacteriol. 192:5848–5849. doi: 10.1128/JB.00951-10.

Huang TW et al. 2012. Complete genome sequence of *Staphylococcus aureus* M013, a pvl-Positive, ST59-SCCmec Type V strain isolated in Taiwan. J. Bacteriol. 194:1256–1257. doi: 10.1128/JB.06666-11.

Köser CU et al. 2012. Rapid whole-genome sequencing for investigation of a neonatal MRSA outbreak. N. Engl. J. Med. 366:2267–2275. doi: 10.1056/NEJMoa1109910.

Kuroda M et al. 2001. Whole genome sequencing of meticillin-resistant *Staphylococcus aureus*. Lancet. 357:1225–1240.

Larner-Svensson H et al. 2013. Complete genome sequence of *Staphylococcus aureus* strain M1, a unique t024-ST8-IVa Danish methicillin-resistant *S. aureus* clone. Genome Announc. 1:e00336–13. doi: 10.1128/genomeA.00336-13.

Li Y et al. 2011. Complete genome sequence of *Staphylococcus aureus* T0131, an ST239-MRSA-SCCmec type III clone isolated in China. J. Bacteriol. 193:3411–3412. doi: 10.1128/JB.05135-11.

Lindqvist M, Isaksson B, Grub C, Jonassen TØ, Hällgren A. 2012. Detection and characterisation of SCCmec remnants in multiresistant methicillin-susceptible *Staphylococcus aureus* causing a clonal outbreak in a Swedish county. Eur. J. Clin. Microbiol. Infect. Dis. 31:141–147. doi: 10.1007/s10096-011-1286-y.

Lowder BV et al. 2009. Recent human-to-poultry host jump, adaptation, and pandemic spread of *Staphylococcus aureus*. Proc. Natl. Acad. Sci. U.S.A. 106:19545–19550. doi: 10.1073/pnas.0909285106.

Mwangi MM et al. 2007. Tracking the in vivo evolution of multidrug resistance in *Staphylococcus aureus* by whole-genome sequencing. Proc. Natl. Acad. Sci. U.S.A. 104:9451–9456. doi: 10.1073/pnas.0609839104.

Neoh H-M et al. 2008. Mutated response regulator graR is responsible for phenotypic conversion of *Staphylococcus aureus* from heterogeneous vancomycin-intermediate resistance to vancomycin-intermediate resistance. Antimicrob. Agents Chemother. 52:45–53. doi: 10.1128/AAC.00534-07.

Nübel U et al. 2010. A timescale for evolution, population expansion, and spatial spread of an emerging clone of methicillin-resistant *Staphylococcus aureus*. PLoS Pathog. 6:e1000855. doi: 10.1371/journal.ppat.1000855.

Richter SS et al. 2011. Detection of *Staphylococcus aureus* isolates with heterogeneous intermediate-level resistance to vancomycin in the United States. J. Clin. Microbiol. 49:4203–4207. doi: 10.1128/JCM.01152-11.

Sass P et al. 2012. Genome sequence of *Staphylococcus aureus* VC40, a vancomycin- and daptomycin-resistant strain, to study the genetics of development of resistance to currently applied last-resort antibiotics. J. Bacteriol. 194:2107–2108. doi: 10.1128/JB.06631-11.

Satola SW et al. 2011. Clinical and laboratory characteristics of invasive infections due to methicillin-resistant *Staphylococcus aureus* isolates demonstrating a vancomycin MIC of 2 micrograms per milliliter: lack of effect of heteroresistant vancomycin-intermediate *S. aureus* phenotype. J. Clin. Microbiol. 49:1583–1587. doi: 10.1128/JCM.01719-10.

Satola SW, Caliendo AM, Farley MM, Patel JB, Burd EM. 2009. Lack of heteroresistance among *Staphylococcus aureus* isolates with vancomycin MICs of 2 micrograms per milliliter by automated testing. J. Clin. Microbiol. 47:2680–2681. doi: 10.1128/JCM.01184-09.

Schijffelen MJ, Boel CHE, van Strijp JAG, Fluit AC. 2010. Whole genome analysis of a livestock-associated methicillin-resistant *Staphylococcus aureus* ST398 isolate from a case of human endocarditis. BMC Genomics. 11:376. doi: 10.1186/1471-2164-11-376.

Stegger M et al. 2012. Genome sequence of *Staphylococcus aureus* strain 11819-97, an ST80-IV European community-acquired methicillin-resistant isolate. J. Bacteriol. 194:1625–1626. doi: 10.1128/JB.06653-11.

Stegger M et al. 2013. Genome sequence of *Staphylococcus aureus* strain CA-347, a USA600 methicillin-resistant isolate. Genome Announc. 1:e00517–13. doi: 10.1128/genomeA.00517-13.

Uhlemann A-C et al. 2012. Identification of a highly transmissible animal-independent *Staphylococcus aureus* ST398 clone with distinct genomic and cell adhesion properties. MBio. 3:e00027–12. doi: 10.1128/mBio.00027-12.

Vogel V, Falquet L, Calderon-Copete SP, Basset P, Blanc DS. 2012. Short term evolution of a highly transmissible methicillin-resistant *Staphylococcus aureus* clone (ST228) in a tertiary care hospital. PLoS ONE. 7:e38969. doi: 10.1371/journal.pone.0038969.

**Table S2:** GenBank ID of the completed *Staphylococcus aureus* genomes included in this study

| **S. No.** | **GenBank ID** | **Strain Name** | **MLST** | **Reference** |
| --- | --- | --- | --- | --- |
| 1 | NC_002953 | MSSA476 | 1 | (Holden et al. 2004) |
| 2 | NC_003923 | MW2 | 1 | (Baba et al. 2002) |
| 3 | NC_002745 | N315 | 5 | (Kuroda et al. 2001) |
| 4 | NC_013450 | ED98 | 5 | (Lowder et al. 2009) |
| 5 | NC_009782 | Mu3 | 5 | (Neoh et al. 2008) |
| 6 | NC_002758 | Mu50 | 5 | (Kuroda et al. 2001) |
| 7 | NC_007793 | USA300_FPR3757 | 8 | (Diep et al. 2006) |
| 8 | NC_021059 | M1 | 8 | (Larner-Svensson et al. 2013) |
| 9 | NC_007795 | NCTC8325 | 8 | (Gillaspy et al. 2006) |
| 10 | NC_009641 | Newman | 8 | (Baba et al. 2008) |
| 11 | NC_010079 | TCH1516 | 8 | (Highlander et al. 2007) |
| 12 | NC_002952 | MRSA252 | 36 | (Holden et al. 2004) |
| 13 | NC_016928 | M013 | 59 | (Huang et al. 2012) |
| 14 | NC_022443 | SA40 | 59 | (Chih-Jung Chen et al. 2013a) |
| 15 | NC_022442 | SA957 | 59 | (Chih-Jung Chen et al. 2013a) |
| 16 | NC_022226 | CN1 | 72 | (Yan Chen et al. 2013c) |
| 17 | NC_017351 | 11819-97 | 80 | (Stegger et al. 2012) |
| 18 | NC_017338 | JKD6159 | 93 | (Chua et al. 2010) |
| 19 | NC_009632 | JH1 | 105 | (Mwangi et al. 2007) |
| 20 | NC_009487 | JH9 | 105 | (Mwangi et al. 2007) |
| 21 | NC_017337 | ED133 | 133 | (Guinane et al. 2010) |
| 22 | NC_007622 | RF122 | 151 | (Herron-Olson et al. 2007) |
| 23 | NC_017340 | 04-02981 | 225 | (Nübel et al. 2010) |
| 24 | NC_020529 | 10388 | 228 | (Vogel et al. 2012) |
| 25 | NC_020564 | 10497 | 228 | (Vogel et al. 2012) |
| 26 | NC_020532 | 15532 | 228 | (Vogel et al. 2012) |
| 27 | NC_020536 | 18341 | 228 | (Vogel et al. 2012) |
| 28 | NC_020537 | 18412 | 228 | (Vogel et al. 2012) |
| 29 | NC_020533 | 16035 | 228 | (Vogel et al. 2012) |
| 30 | NC_020566 | 16125 | 228 | (Vogel et al. 2012) |
| 31 | NC_020568 | 18583 | 228 | (Vogel et al. 2012) |
| 32 | NC_017341 | JKD6008 | 239 | (Howden et al. 2010) |
| 33 | NC_017347 | T0131 | 239 | (Li et al. 2011) |
| 34 | NC_017331 | TW20 | 239 | (Holden et al. 2010) |
| 35 | NC_002951 | COL | 250 | (Gill et al. 2005) |
| 36 | NC_017673 | 71193 | 398 | (Uhlemann et al. 2012) |
| 37 | NC_018608 | 08BA02176 | 398 | (Golding et al. 2012) |
| 38 | NC_017333 | ST398 | 398 | (Schijffelen et al. 2010) |
| 39 | NC_017349 | LGA251 | 425 | (García-Álvarez et al. 2011) |
| 40 | NC_016941 | MSHR1132 | 1850 | (Holt et al. 2011) |
| 41 | NC_022222 | 6850 | 50 | Unpublished |
| 42 | NC_022113 | 55-2053 | 30 | Unpublished |
| 43 | NC_021670 | Bmb9393 | 239 | (Costa et al. 2013) |
| 44 | NC_021554 | CA-347 | 45 | (Stegger et al. 2013) |
| 45 | NC_017343 | ECT-R 2 | 5 | (Lindqvist et al. 2012) |
| 46 | NC_017763 | HO 5096 0412 | 22 | (Köser et al. 2012) |
| 47 | NC_016912 | VC40 | 8 | (Sass et al. 2012) |
| 48 | NC_022604 | Z172 | 239 | (Feng Chen et al. 2013b) |
| 49 | NC_017342 | TCH60 | 30 | Unpublished |

**References**

Baba T et al. 2002. Genome and virulence determinants of high virulence community-acquired MRSA. Lancet. 359:1819–1827.

Baba T, Bae T, Schneewind O, Takeuchi F, Hiramatsu K. 2008. Genome sequence of *Staphylococcus aureus* strain Newman and comparative analysis of staphylococcal genomes: polymorphism and evolution of two major pathogenicity islands. J. Bacteriol. 190:300–310. doi: 10.1128/JB.01000-07.

Chen C-J et al. 2013a. Characterization and comparison of 2 distinct epidemic community-associated methicillin-resistant *Staphylococcus aureus* clones of ST59 lineage. PLoS ONE. 8:e63210. doi: 10.1371/journal.pone.0063210.

Chen F, Lauderdale T, Wang L, Huang I. 2013b. Complete genome sequence of *Staphylococcus aureus* Z172, a vancomycin-intermediate and daptomycin-nonsusceptible methicillin-resistant strain isolated in Taiwan. Genome Announc. 1:e01011–13. doi: 10.1128/genomeA.01011-13.

Chen Y, Chatterjee SS, Porcella SF, Yu Y-S, Otto M. 2013c. Complete genome sequence of a Pantón-Valentine leukocidin-negative community-associated methicillin-resistant *Staphylococcus aureus* strain of sequence type 72 from Korea. PLoS ONE. 8:e72803. doi: 10.1371/journal.pone.0072803.

Chua K et al. 2010. Complete genome sequence of *Staphylococcus aureus* strain JKD6159, a unique Australian clone of ST93-IV community methicillin-resistant *Staphylococcus aureus*. J. Bacteriol. 192:5556–5557. doi: 10.1128/JB.00878-10.

Costa MOC et al. 2013. Complete genome sequence of a variant of the methicillin-resistant *Staphylococcus aureus* ST239 lineage, strain BMB9393, displaying superior ability to accumulate ica-independent biofilm. Genome Announc. 1:e00576–13. doi: 10.1128/genomeA.00576-13.

Diep BA et al. 2006. Complete genome sequence of USA300, an epidemic clone of community-acquired meticillin-resistant *Staphylococcus aureus*. The Lancet. 367:731–739.

Fritz SA et al. 2011. Effectiveness of measures to eradicate *Staphylococcus aureus* carriage in patients with community-associated skin and soft-tissue infections: a randomized trial. Infect Control Hosp Epidemiol. 32:872–880. doi: 10.1086/661285.

García-Álvarez L et al. 2011. Meticillin-resistant *Staphylococcus aureus* with a novel mecA homologue in human and bovine populations in the UK and Denmark: a descriptive study. Lancet Infect Dis. 11:595–603. doi: 10.1016/S1473-3099(11)70126-8.

Gill SR et al. 2005. Insights on evolution of virulence and resistance from the complete genome analysis of an early methicillin-resistant *Staphylococcus aureus* strain and a biofilm-producing methicillin-resistant *Staphylococcus epidermidis* strain. J. Bacteriol. 187:2426–2438. doi: 10.1128/JB.187.7.2426-2438.2005.

Gillaspy AF et al. 2006. The *Staphylococcus aureus* NCTC 8325 genome. In Gram-Positive Pathogens, pp. 381–412. Edited by V. A. Fischetti, R. P. Novick, J. J. Ferretti, D. A. Portnoy & J. I. Rood. Washington, DC: American Society for Microbiology.

Golding GR et al. 2012. Whole-genome sequence of livestock-associated st398 methicillin-resistant *Staphylococcus aureus* isolated from humans in Canada. J. Bacteriol. 194:6627–6628. doi: 10.1128/JB.01680-12.

Guinane CM et al. 2010. Evolutionary genomics of *Staphylococcus aureus* reveals insights into the origin and molecular basis of ruminant host adaptation. Genome Biology and Evolution. 2:454–466. doi: 10.1093/gbe/evq031.

Herron-Olson L, Fitzgerald JR, Musser JM, Kapur V. 2007. Molecular correlates of host specialization in *Staphylococcus aureus*. PLoS ONE. 2:e1120. doi: 10.1371/journal.pone.0001120.

Highlander SK et al. 2007. Subtle genetic changes enhance virulence of methicillin resistant and sensitive *Staphylococcus aureus*. BMC Microbiol. 7:99. doi: 10.1186/1471-2180-7-99.

Holden MTG et al. 2004. Complete genomes of two clinical *Staphylococcus aureus* strains: evidence for the rapid evolution of virulence and drug resistance. Proc. Natl. Acad. Sci. U.S.A. 101:9786–9791. doi: 10.1073/pnas.0402521101.

Holden MTG et al. 2010. Genome sequence of a recently emerged, highly transmissible, multi-antibiotic- and antiseptic-resistant variant of methicillin-resistant *Staphylococcus aureus* , sequence type 239 (TW). J. Bacteriol. 192:888–892.

Holt DC et al. 2011. A very early-branching *Staphylococcus aureus* lineage lacking the carotenoid pigment staphyloxanthin. Genome Biology and Evolution. 3:881–895. doi: 10.1093/gbe/evr078.

Howden BP et al. 2010. Complete genome sequence of Staphylococcus aureus strain JKD6008, an ST239 clone of methicillin-resistant Staphylococcus aureus with intermediate-level vancomycin resistance. J. Bacteriol. 192:5848–5849. doi: 10.1128/JB.00951-10.

Huang TW et al. 2012. Complete genome sequence of *Staphylococcus aureus* M013, a pvl-Positive, ST59-SCCmec Type V strain isolated in Taiwan. J. Bacteriol. 194:1256–1257. doi: 10.1128/JB.06666-11.

Köser CU et al. 2012. Rapid whole-genome sequencing for investigation of a neonatal MRSA outbreak. N. Engl. J. Med. 366:2267–2275. doi: 10.1056/NEJMoa1109910.

Kuroda M et al. 2001. Whole genome sequencing of meticillin-resistant *Staphylococcus aureus*. Lancet. 357:1225–1240.

Larner-Svensson H et al. 2013. Complete genome sequence of *Staphylococcus aureus* strain M1, a unique t024-ST8-IVa Danish methicillin-resistant *S. aureus* clone. Genome Announc. 1:e00336–13. doi: 10.1128/genomeA.00336-13.

Li Y et al. 2011. Complete genome sequence of *Staphylococcus aureus* T0131, an ST239-MRSA-SCCmec type III clone isolated in China. J. Bacteriol. 193:3411–3412. doi: 10.1128/JB.05135-11.

Lindqvist M, Isaksson B, Grub C, Jonassen TØ, Hällgren A. 2012. Detection and characterisation of SCCmec remnants in multiresistant methicillin-susceptible *Staphylococcus aureus* causing a clonal outbreak in a Swedish county. Eur. J. Clin. Microbiol. Infect. Dis. 31:141–147. doi: 10.1007/s10096-011-1286-y.

Lowder BV et al. 2009. Recent human-to-poultry host jump, adaptation, and pandemic spread of *Staphylococcus aureus*. Proc. Natl. Acad. Sci. U.S.A. 106:19545–19550. doi: 10.1073/pnas.0909285106.

Mwangi MM et al. 2007. Tracking the in vivo evolution of multidrug resistance in *Staphylococcus aureus* by whole-genome sequencing. Proc. Natl. Acad. Sci. U.S.A. 104:9451–9456. doi: 10.1073/pnas.0609839104.

Neoh H-M et al. 2008. Mutated response regulator graR is responsible for phenotypic conversion of *Staphylococcus aureus* from heterogeneous vancomycin-intermediate resistance to vancomycin-intermediate resistance. Antimicrob. Agents Chemother. 52:45–53. doi: 10.1128/AAC.00534-07.

Nübel U et al. 2010. A timescale for evolution, population expansion, and spatial spread of an emerging clone of methicillin-resistant *Staphylococcus aureus*. PLoS Pathog. 6:e1000855. doi: 10.1371/journal.ppat.1000855.

Richter SS et al. 2011. Detection of *Staphylococcus aureus* isolates with heterogeneous intermediate-level resistance to vancomycin in the United States. J. Clin. Microbiol. 49:4203–4207. doi: 10.1128/JCM.01152-11.

Sass P et al. 2012. Genome sequence of *Staphylococcus aureus* VC40, a vancomycin- and daptomycin-resistant strain, to study the genetics of development of resistance to currently applied last-resort antibiotics. J. Bacteriol. 194:2107–2108. doi: 10.1128/JB.06631-11.

Satola SW et al. 2011. Clinical and laboratory characteristics of invasive infections due to methicillin-resistant *Staphylococcus aureus* isolates demonstrating a vancomycin MIC of 2 micrograms per milliliter: lack of effect of heteroresistant vancomycin-intermediate *S. aureus* phenotype. J. Clin. Microbiol. 49:1583–1587. doi: 10.1128/JCM.01719-10.

Satola SW, Caliendo AM, Farley MM, Patel JB, Burd EM. 2009. Lack of heteroresistance among *Staphylococcus aureus* isolates with vancomycin MICs of 2 micrograms per milliliter by automated testing. J. Clin. Microbiol. 47:2680–2681. doi: 10.1128/JCM.01184-09.

Schijffelen MJ, Boel CHE, van Strijp JAG, Fluit AC. 2010. Whole genome analysis of a livestock-associated methicillin-resistant *Staphylococcus aureus* ST398 isolate from a case of human endocarditis. BMC Genomics. 11:376. doi: 10.1186/1471-2164-11-376.

Stegger M et al. 2012. Genome sequence of *Staphylococcus aureus* strain 11819-97, an ST80-IV European community-acquired methicillin-resistant isolate. J. Bacteriol. 194:1625–1626. doi: 10.1128/JB.06653-11.

Stegger M et al. 2013. Genome sequence of *Staphylococcus aureus* strain CA-347, a USA600 methicillin-resistant isolate. Genome Announc. 1:e00517–13. doi: 10.1128/genomeA.00517-13.

Uhlemann A-C et al. 2012. Identification of a highly transmissible animal-independent *Staphylococcus aureus* ST398 clone with distinct genomic and cell adhesion properties. MBio. 3:e00027–12. doi: 10.1128/mBio.00027-12.

Vogel V, Falquet L, Calderon-Copete SP, Basset P, Blanc DS. 2012. Short term evolution of a highly transmissible methicillin-resistant *Staphylococcus aureus* clone (ST228) in a tertiary care hospital. PLoS ONE. 7:e38969. doi: 10.1371/journal.pone.0038969.

**Table S3:** SNPs found within the most common VISA associated genes.

*Note:* The MIC and VSSA/VISA classification is based on Etest. The amino acid highlighted in red has also been reported in earlier studies. The locus tag, codon and bp coordinates are according to N315 reference genome.

**Table S4:** rpoB mutations in the rifampin-resistant *Staphylococcus aureus* isolates^a^

|  | GA984 | SR2609 | SR4035 | MN105 | SR1287 | SR220 | 407 | 126 | 312 | 115 | 109 | 504 | 123 |
| --- | --- | --- | --- | --- | --- | --- | --- | --- | --- | --- | --- | --- | --- |
| BMD MIC | 2 | ≤2 | ≤2 | 2 | ≤2 | ≤2 | 4 | 4 | 4 | 4 | 4 | 4 | 4 |
| Etest MIC | 2 | 2 | 2 | 3 | 3 | 3 | 3 | 4 | 4 | 4 | 6 | 6 | 6 |
| PAP-AUC ratio | 0.9 | 0.89 | 1.04 | 1.05 | 1.14 | 1.03 | 1.73 | 1.65 | 1.97 | 1.74 | 1.8 | 1.58 | 2 |
| Rifampin profile | R | R | I | R | R | R | R | R | R | I | R | R | R |
| **rpoB SNPs** |  |  |  |  |  |  |  |  |  |  |  |  |  |
| A2T | - | - | - | - | - | - | - | - | - | - | - | - | - |
| V96M | - | - | - | - | - | M | - | - | - | - | - | - | - |
| S203N | - | - | - | - | - | - | - | - | - | - | - | - | - |
| P293L | - | - | - | L | - | - | - | - | - | - | - | - | - |
| D320N | - | - | - | - | - | - | - | - | N | - | - | - | - |
| V411A | - | - | - | - | - | - | - | - | - | - | - | - | - |
| L415P | - | - | - | - | - | P | - | - | - | - | - | - | - |
| S464P | - | - | - | - | - | - | - | - | - | - | P | - | - |
| D471G/N | N | G | - | - | - | - | - | - | - | - | - | - | - |
| N474K | - | - | K | - | - | - | - | - | - | - | - | - | - |
| A477D | - | - | - | - | - | - | - | - | - | - | - | - | - |
| H481Y/L/N | - | - | - | Y | Y | L | Y | Y | Y | N | - | Y | Y |
| L485P | - | - | - | - | P | - | - | - | - | - | - | - | - |
| P519L | - | L | - | - | - | - | - | - | - | - | - | - | - |
| N523D | D | - | - | - | - | - | - | - | - | - | - | - | - |
| H861D | - | - | - | - | - | - | - | - | - | - | - | - | - |
| G865S | - | - | - | - | - | S | - | - | - | - | - | - | - |

^a^The rpoB mutation in 13 rifampin-resistant isolates. I, Intermediate level resistant; R, High level resistant. Dash represents allele identical to the N315 reference sequence. Alternate alleles are indicated.
